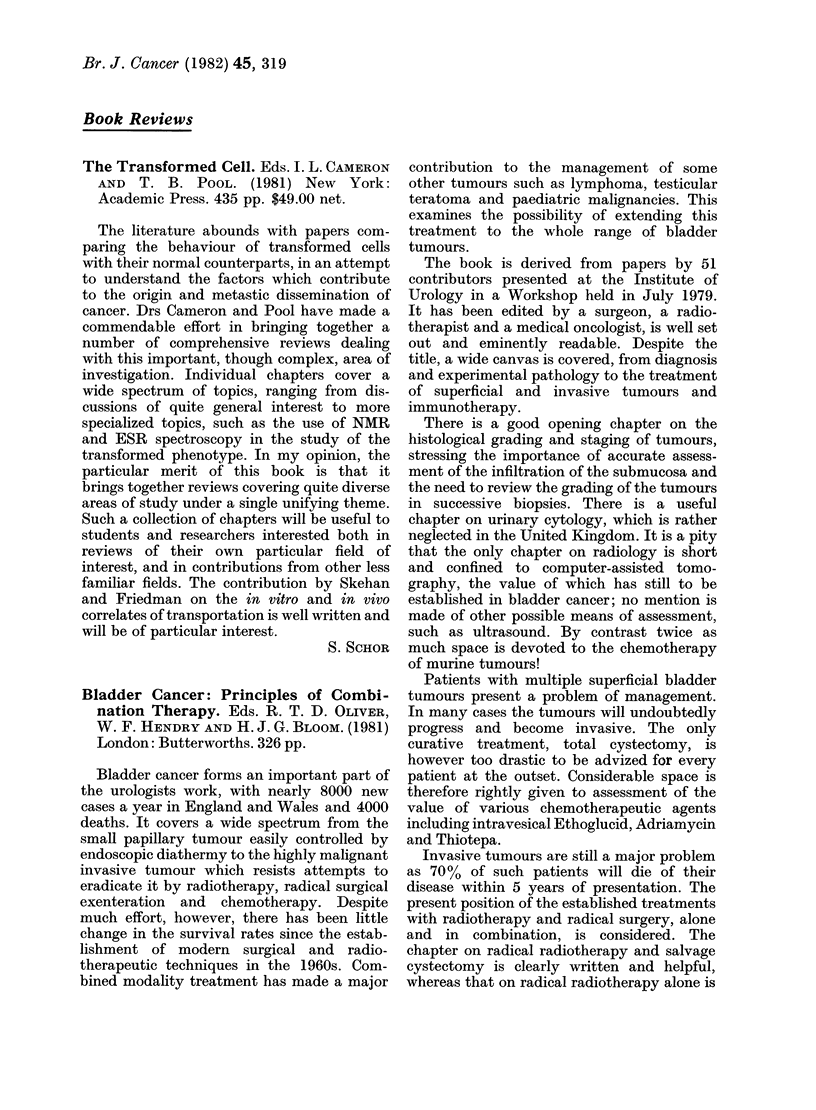# The Transformed Cell

**Published:** 1982-02

**Authors:** S. Schor


*Br. J. Cancer* (1982) **45**, 319

### *Book Reviews*

**The Transformed Cell.** Eds. I. L. CAMERON AND T. B. POOL. (1981) New York: Academic Press. 435 pp. $49.00 net.

The literature abounds with papers comparing the behaviour of transformed cells with their normal counterparts, in an attempt to understand the factors which contribute to the origin and metastic dissemination of cancer. Drs Cameron and Pool have made a commendable effort in bringing together a number of comprehensive reviews dealing with this important, though complex, area of investigation. Individual chapters cover a wide spectrum of topics, ranging from discussions of quite general interest to more specialized topics, such as the use of NMR and ESR spectroscopy in the study of the transformed phenotype. In my opinion, the particular merit of this book is that it brings together reviews covering quite diverse areas of study under a single unifying theme. Such a collection of chapters will be useful to students and researchers interested both in reviews of their own particular field of interest, and in contributions from other less familiar fields. The contribution by Skehan and Friedman on the *in vitro* and *in vivo* correlates of transportation is well written and will be of particular interest.

S. SCHOR